# Sarcopenia Identification Using Alternative Vertebral Landmarks in Individuals with Lung Cancer

**DOI:** 10.3390/muscles3020012

**Published:** 2024-04-16

**Authors:** Cecily A. Byrne, Giamila Fantuzzi, Jeremy T. Stephan, Sage Kim, Vanessa M. Oddo, Timothy J. Koh, Sandra L. Gomez

**Affiliations:** 1 Cancer Health Equity and Career Development Program, University of Illinois Chicago, 1747 W. Roosevelt Rd., Chicago, IL 60608, USA; 2 Department of Kinesiology and Nutrition, University of Illinois Chicago, 1919 W. Taylor St., Chicago, IL 60612, USA; 3 Department of Radiology, Rush University Medical Center, 1653 W. Congress Parkway, Chicago, IL 60612, USA; 4 School of Public Health, University of Illinois Chicago, 1603 W. Taylor St., Chicago, IL 60612, USA; 5 Department of Clinical Nutrition, Rush University, 600 S. Paulina St., Chicago, IL 60612, USA

**Keywords:** sarcopenia, lung cancer, alternative vertebral landmarks, agreement

## Abstract

(1) Background: Sarcopenia, or low skeletal mass index (SMI), contributes to higher lung cancer mortality. The SMI at third lumbar vertebrae (L3) is the reference standard for body composition analysis. However, there is a need to explore the validity of alternative landmarks in this population. We compared the agreement of sarcopenia identification at the first lumbar (L1) and second lumbar (L2) to L3 in non-Hispanic Black (NHB) and White (NHW) individuals with lung cancer. (2) Methods: This retrospective, cross-sectional study included 214 NHB and NHW adults with lung cancer. CT scans were analyzed to calculate the SMI at L1, L2, and L3. T-tests, chi-square, Pearson’s correlation, Cohen’s kappa, sensitivity, and specificity analysis were used. (3) Results: Subjects presented with a mean age of 68.4 ± 9.9 years and BMI of 26.3 ± 6.0 kg/m^2^. Sarcopenia prevalence varied from 19.6% at L1 to 39.7% at L3. Cohen’s kappa coefficient was 0.46 for L1 and 0.64 for L2, indicating weak and moderate agreement for the identification of sarcopenia compared to L3. (4) Conclusions: Sarcopenia prevalence varied greatly depending on the vertebral landmark used for assessment. Using L2 or L1 alone resulted in a 16.8% and 23.8% misclassification of sarcopenia in this cohort of individuals with lung cancer.

## 1. Introduction

Sarcopenia, or low muscle strength and quantity or quality [1], is associated with poor prognosis, including reduced progression-free and overall survival in lung cancer [2,3,4,5]. Clinical computed tomography (CT) scans can be used to assess muscle quantity or quality (but not muscle strength) in individuals with cancer, which is referred to as CT-assessed sarcopenia. The muscle mass at the third lumbar vertebra (L3) using abdominal CT scans is the reference standard for body composition analysis, as skeletal muscle and adipose tissue in this region are highly correlated with total body skeletal muscle (0.92–0.94, *p* < 0.001) and adipose tissue mass in healthy individuals and in those with lung or colorectal cancer [6,7]. However, analyzing CT scans at L3 is not always feasible for patients with lung cancer, as most individuals with lung cancer receive chest CT scans initially, and the scanning area does not always extend to L3 [8,9]. A recent systematic review investigating the use of alternative vertebral landmarks to L3 for skeletal muscle evaluation indicated the lack of an abdominal CT scan as the primary reason for the use of alternative vertebral landmarks [10]. Previous studies in healthy adults [6,11] and in those with lung cancer [8,9] indicate that skeletal muscle at the first lumbar vertebra (L1) or the second lumbar vertebra (L2) may be appropriate alternatives to L3 for sarcopenia analysis as they are highly correlated with the reference landmark. L1 was available in 94% of chest CT scans from different institutions [9], whereas the use of L2 has not be evaluated specifically for individuals with lung cancer. Therefore, evaluating the use of L1 and L2 for sarcopenia assessment compared to L3 would contribute to the limited evidence in the use of alternative lumbar vertebral landmarks in individuals with lung cancer.

Body composition differs by race and ethnicity with non-Hispanic Black (NHB) individuals having a higher muscle mass across the lifespan and a similar BMI compared to non-Hispanic White (NHW) individuals [12,13]. In cancer, NHB individuals have a lower prevalence of sarcopenia [14,15,16] compared to NHW individuals. However, the current published sex-specific cut-off values for defining sarcopenia in cancer at L3 [7,17,18,19] and at L1 [8] are based on presumably NHW and Asian populations, respectively, as race/ethnicity was not disclosed in the studies. Moreover, in healthy adults, the sex-specific healthy reference mean SMI and the cut-offs for sarcopenia identification at vertebral levels ranging from the 10th thoracic (T10) to the 5th lumbar (L5) are based on groups that may not be reflective of diverse racial and ethnic populations [11]. Therefore, it is not known if the current sex-specific cut-off values for defining sarcopenia are valid in diverse populations.

To date, limited studies have investigated the agreement of sarcopenia identification using multiple vertebral landmarks compared to L3 in individuals with lung cancer, particularly in diverse populations. Given the prognostic implications of sarcopenia in lung cancer, we used clinical CT scans to (1) explore the prevalence of CT-assessed sarcopenia in NHB and NHW individuals with lung cancer at three vertebral landmarks using published sex-specific cut-off values and (2) compare the agreement of sarcopenia identification at L1 and L2 to L3 in individuals with lung cancer. This research will help us to determine the extent of sarcopenia misclassification when L1 or L2 is used for sarcopenia identification in lieu of L3 in a population that includes an equal representation of NHB and NHW individuals.

## 2. Results

### 2.1. Overall Demographics of Subjects

The sample consisted of an equal number of males (n = 107) and females (n = 107) with a mean age of 68.4 ± 9.9 years and a BMI of 26.3 ± 6.0 kg/m^2^ (Table 1). Half of the subjects were NHB, and half were NHW individuals. A higher percent of subjects had late-stage cancer (62.1%) compared to early-stage cancer (37.9%; *p* < 0.001), with significantly more NHB individuals (73.8%) presenting with late-stage cancer compared to NHW individuals (50.5%; *p* < 0.001) and no differences by sex (Table 1). The majority of subjects had a current/former history of smoking with more females having never smoked compared to males (*p* = 0.01; Table 1).

### 2.2. Assessing the Use of Alternative Vertebral Landmarks

Sarcopenia assessment using alternative vertebral landmarks depends on the availability of the muscle cross-sectional area and the correlation of the SMI at this alternate landmark to the reference standard (L3). In our cohort, L1 was present in 88% (258/293) of CT scans, including chest, whole body, and/or chest/abdominal/pelvis scans. A total of 84% (246/293) and 74% (218/293) of subjects had a muscle cross-sectional area available at L2 and L3, respectively. Of those individuals with only a chest CT (n = 52), the L1 muscle cross-sectional area was available from 65.4% of the scans (34/52), L2 from 51.9% of the scans (27/52), and L3 from 21.2% of the scans (11/52).

As in the previously published literature [8,11,20], the correlation of the L3-derived SMI with both L1 (r = 0.89; *p* < 0.0001) and L2 (r = 0.93; *p* < 0.0001) was high in our sample. When stratifying by sex and race, the correlations between L1 and L3 and between L2 and L3 were similar (*p* < 0.0001).

### 2.3. Comparison of Sarcopenia Prevalence at L1, L2, and L3

Using the Derstine et al. criteria for sarcopenia diagnosis [11], the prevalence of sarcopenia was 19.6% at L1, 31.3% at L2, and 39.7% at L3 (Table 2). While it did not achieve statistical significance (*p* = 0.05), the age was higher in those with sarcopenia (70.0 ± 10.2 years) than those without sarcopenia (67.4 ± 9.5 years) at L3. Height was higher and weight was lower in individuals with sarcopenia compared to those without sarcopenia at all three landmarks (Table 2). Evaluating the correlation between height and the SMI, height was negatively correlated with the SMI in females only at all three vertebral landmarks (r = −0.20, *p* = 0.04 at L1; r = −0.21, *p* = 0.03 at L2; r = −0.23, *p* = 0.02 at L3). Height was nearly correlated with the SMI at L2 in males (r = −0.18, *p* = 0.06) but did not reach statistical significance at L1 or L3. The SMI was positively correlated with the BMI in both males and females at all three vertebral landmarks (males = r = 0.68, *p* < 0.0001 at L1; r = 0.60, *p* < 0.0001 at L2; r = 0.61, *p* < 0.0001 at L3; females = r = 0.66, *p* < 0.0001 at L1; r = 0.62, *p* < 0.0001 at L2; r = 0.54, *p* < 0.0001 at L3). The BMI was lower in those with sarcopenia compared to those without sarcopenia at all three landmarks (*p* < 0.0001 at L1, L2, and L3; Table 2). Sarcopenia was more prevalent in males compared to females at all three landmarks, but no differences in sarcopenia prevalence were found by race and ethnicity (Table 2). In those with sarcopenia, late-stage lung cancer was more prevalent compared to early-stage lung cancer at all three vertebral landmarks (*p* = 0.0007 at L1; *p* = 0.001 at L2; *p* = 0.003 at L3; Table 2).

### 2.4. Agreement of Sarcopenia Identification at Alternative Vertebral Landmarks

The agreement for the identification of sarcopenia at L1 and L2 was compared to L3. Cohen’s kappa coefficient was 0.46 for L1, indicating weak agreement with L3 (Table 3) [21]. The overall accuracy for the classification of sarcopenia status was 76.2% for L1 compared to L3, indicating 23.8% of individuals in the sample were misclassified according to sarcopenia status. Sensitivity for L1 was 44.7%, and specificity was 96.9% for detecting sarcopenia status compared to L3. The positive predictive value (true positives) of L1 was 90.5%, and the negative predictive value was 72.7% for L1. The area under the curve summarized the overall accuracy of L1 (0.71) to identify sarcopenia compared to L3, indicating an acceptable ability to distinguish between those with and without sarcopenia [22].

Cohen’s kappa coefficient was 0.64 for L2, indicating moderate agreement [21] with L3 (Table 3). The overall accuracy for the classification of sarcopenia status was 84.2% for L2 compared to L3, which indicates 16.8% of individuals in this sample were misclassified according to sarcopenia status when using L2 for assessment. Sensitivity was 68.2% and specificity was 93% for L2 (Table 3) to detect sarcopenia status. The positive predictive value was 86.6% for L2, and the negative predictive value was 81.6% (Table 3). The area under the curve summarized the overall accuracy of L2 (0.81) to identify sarcopenia compared to L3, indicating an acceptable ability to distinguish between those with and without sarcopenia [22].

Sarcopenia was identified at all three vertebral landmarks in 16.4% of subjects (n = 35; Group 1), whereas no sarcopenia was identified at all three landmarks in 55.6% of subjects (n = 119; Group 2; Table 4). When comparing Group 1 and 2, the height was higher (*p* = 0.003), weight was lower (*p* < 0.0001), and BMI was lower (*p* < 0.0001) in Group 1 compared to Group 2 (Table 4). There were also more males in Group 1 where all three landmarks identified sarcopenia and more females in Group 2 where all three landmarks identified no sarcopenia (*p* < 0.001; Table 4).

**Figure 1:**
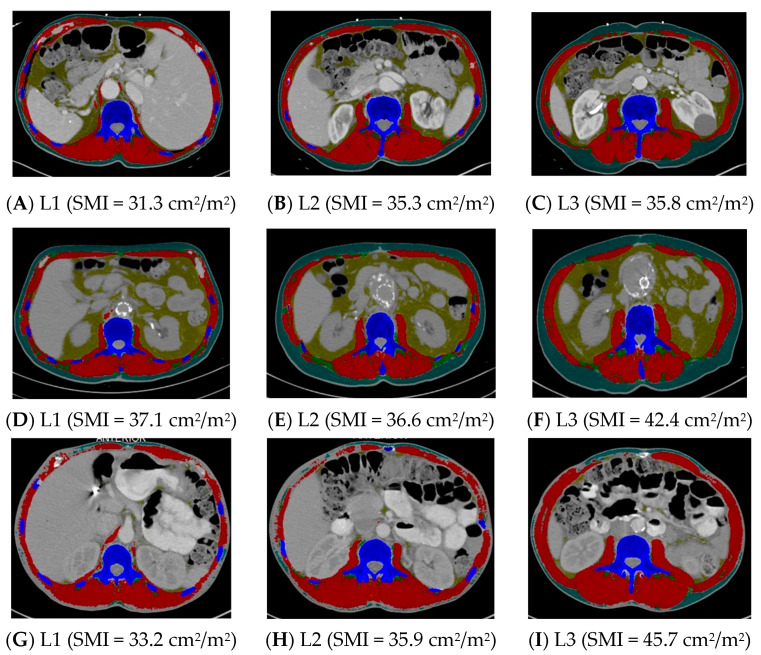
Computed tomography images demonstrating consistency and inconsistency amongst the three vertebral landmarks for the identification of sarcopenia. Images (**A**–**C**) consistently identify sarcopenia, whereas images (**D**–**I**) inconsistently identify sarcopenia. L1 = first lumbar, L2 = second lumbar, L3 = third lumbar, SMI = skeletal mass index, cm = centimeters, m = meters. Skeletal muscle and adipose tissues were tagged by ABACS+; red= skeletal muscle; yellow= visceral adipose tissue; green= intermuscular adipose tissue; teal= subcutaneous adipose tissue; blue= bone.

Group 1 (n = 35) was compared to Group 3, where at least one landmark was inconsistent in sarcopenia identification (n = 60) (Table 4). The height did not differ between these two groups, but the weight was lower and the BMI was lower in Group 1 compared to Group 3 (*p* < 0.0001). Groups 1 and 3 did not differ in age, sex, race and ethnicity, or stage of cancer (Table 4). See Figure 1 for examples of CT scans demonstrating consistency and inconsistency in the identification of sarcopenia across all three landmarks.

## 3. Discussion

This study found that the frequency of sarcopenia in NHB and NHW individuals with lung cancer, as assessed by the sex-specific published cut-off values for the SMI in healthy individuals at L1, L2, and L3 [11], differs greatly depending on the vertebral landmark used for evaluation. The area under the curve for both L1 and L2 indicates an acceptable ability to detect those with and without sarcopenia, and the specificity of both landmarks was high in detecting true negatives or those who do not have sarcopenia. However, the sensitivity, or detecting true positives for sarcopenia, was suboptimal for both landmarks. Given these findings, the use of the SMI at L1 and L2 in individuals with lung cancer may not accurately capture individuals with sarcopenia compared to the use of the L3 reference landmark.

The use of the SMI at L1 and L2 to identify sarcopenia resulted in weak and moderate agreement, respectively, compared to L3. This indicates the detection of sarcopenia may be low when using these alternative landmarks. Specifically, both L1 and L2 SMI were not highly sensitive in identifying individuals who truly had sarcopenia (as assessed at the reference standard L3), indicating these vertebral landmarks may result in false negatives. This study found that roughly 32% and 55% of the 85 subjects with sarcopenia at L3 were not identified as having sarcopenia when using the SMI at L2 and L1, respectively. This is important as sarcopenia leads to decreased overall survival and progression-free survival in lung cancer [2,3,4,5], and accurately identifying individuals at a high risk for mortality and providing appropriate interventions are critical to improving lung cancer outcomes. With weak and moderate agreement in the identification of sarcopenia for L1 and L2 and a suboptimal sensitivity of both landmarks, there may be individuals in need of interventions that are currently going undetected.

However, the SMI at both alternative landmarks were highly specific in identifying those who do not have sarcopenia compared to L3, which may result in fewer false positives and unnecessary interventions. This is consistent with studies by Derstine et al. [11] and Kim et al. [8] where they evaluated the specificity of L1 (both studies) and L2 (Derstine et al. only) to identify an individual without sarcopenia compared to L3. In both studies, the specificity for L1 and L2 to identify no sarcopenia compared to L3 ranged from 99.8 to 100%, which was consistent with the values of 93.0–96.9% for L1 and L2 in this study. In any tool used to screen for a disease or health condition, a test with the highest accuracy and least error is the most desirable with high sensitivity (identify those with the disease correctly) and high specificity (determine those without the disease correctly) [23]. While this may not always be feasible with screening tools, it would be preferable for alternative vertebral landmarks to be accurate and sensitive to detect those who have sarcopenia.

Derstine et al. [11] found an overall accuracy of 99.3% and 99.5% for L1 and L2 cut-offs in healthy females and 98.7% and 99.7% for L1 and L2 cut-offs in healthy males compared to the L3 reference. In addition, Kim et al. [8] found an overall accuracy of 98.9% for L1 SMI compared to L3 SMI cut-offs in individuals with small-cell lung cancer, both of which were higher values compared to the accuracy of 76.2% at L1 and 83.2% at L2 in this study. The overall accuracy differences may be due to the difference in the populations studied (e.g., young, healthy kidney donors [11] versus an Asian population with small-cell lung cancer and a mean BMI of 22.4 ± 3.6 kg/m^2^ [8] versus a population of NHB and NHW individuals with lung cancer and a BMI of 26.3 ± 6.0 kg/m^2^ in this study). Another potential explanation is the low prevalence of sarcopenia altogether (0.3–0.7% in healthy males and females, respectively) as identified at L3 in the Derstine et al. [11] study compared to the sarcopenia rate of 39.7% at L3 in this study of individuals with lung cancer. The prevalence of sarcopenia in the Kim et al. [8] study is unknown. The overall accuracy considers the sensitivity and specificity of the landmark in identifying sarcopenia compared to L3, and with very few young, healthy subjects with sarcopenia at L3 in the Derstine et al. study (three females, one male) [11], the sensitivity will be low and specificity high with a high overall accuracy. In the Derstine et al. study, sensitivity for the identification of sarcopenia at L1 and L2 compared to L3 ranged from 0 to 33%, which the authors attribute to the low number of healthy individuals with sarcopenia overall as assessed at L3 [11]. Derstine et al. also noted the difficulty in using specific cut-off values to determine sarcopenia status, and the use of a continuum may be better to categorize sarcopenia risk [11].

In addition, the current cut-offs for SMI using CT imaging in healthy individuals are based on groups that may not be inclusive of diverse racial and ethnic populations [11]. It is possible that the published sex-specific cut-off values for various landmarks do not accurately capture sarcopenia or low muscle quantity in our sample that consists of 50% NHB individuals. Further research is needed to determine CT-based criteria for a healthy SMI and sarcopenia identification in diverse populations. Additionally, our data indicate a negative correlation between the SMI and height for females, as well as a positive correlation between the SMI and BMI in both males and females. This is consistent with findings by Derstine et al. with similar Pearson correlation coefficients [24]. The use of the SMI and currently published sex-specific cut-offs may bias the results to identify sarcopenia in taller individuals and those with a low BMI [24].

The agreement in the identification of sarcopenia between L1 and L3 was weak, and the accuracy and sensitivity of using L1 to identify sarcopenia were lower compared to L2. This may be due to “increased muscle complexity” at the chest wall and ribs at L1 [25] and may result in a lower ability to identify skeletal muscle at L1. While the muscle cross-sectional area at L1 was the most prevalent data point in this cohort of individuals with lung cancer and highly correlated with the SMI at L3, caution is advised when using solely the SMI at L1 to identify individuals with sarcopenia, as individuals may be misclassified as not having sarcopenia. We recommend further research with a larger and more diverse sample to determine the sensitivity and specificity of using L1 for sarcopenia identification compared to L3. Additionally, with the advent of newer artificial intelligence technologies for body composition analysis, multi-slice selections to identify sarcopenia or volumetric measurements are recommended; however, reference standards for these multi-slice or volumetric measurements are needed to make comparisons across different studies and across different populations.

Only 16% of individuals presented with all three vertebral landmarks identifying sarcopenia (Group 1), while nearly 56% of individuals had all three vertebral landmarks identifying no sarcopenia (Group 2). It is reassuring that the majority (72%) of the individuals were consistently identified by the three landmarks, but this also indicates that 28% were not (Group 3). Group 1 was composed of a majority of males and those with late-stage lung cancer. This is consistent with prior studies demonstrating that males have a higher prevalence of sarcopenia compared to females [9,14,17,26] and that sarcopenia is more prevalent in late-stage cancer compared to early-stage [17,26,27]. Finally, individuals in Group 1 had lower BMI, taller heights, and lower weights compared to Group 2, supporting potential biases in the use of SMI cut-offs to identify sarcopenia in taller individuals with low BMI [24].

## 4. Limitations

This is a cross-sectional study and only investigates sarcopenia prevalence at cancer diagnosis, thus causation cannot be inferred. This study compared sarcopenia identification using alternative vertebral landmarks to the reference standard L3; however, the authors did not assess the agreement of or validate the identification of sarcopenia at any of the vertebral landmarks against whole-body skeletal muscle. In addition, the authors did not compare the mortality rates for those with sarcopenia to those without sarcopenia, which would extend the clinical implications of this research. The subjects were obtained from a single urban facility and may have been biased to individuals who access or receive routine medical care, which may not represent the overall lung cancer population. Finally, the SMI is negatively correlated with height, and positively correlated with the BMI, and the sarcopenia cut-offs may bias the results towards identifying sarcopenia in taller individuals and those with low BMI [24]. In future studies, we will optimize skeletal muscle area adjustment for height and the BMI [24].

## 5. Materials and Methods

### 5.1. Study Population and Setting

**Figure 2:**
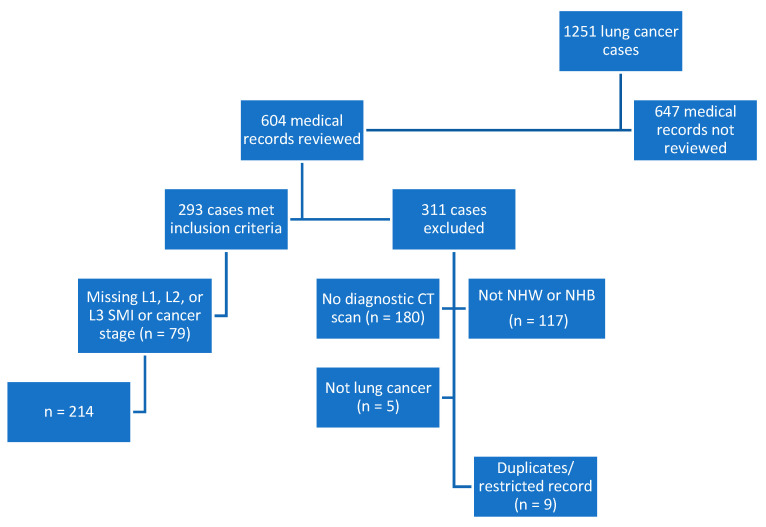
Flowchart depicting study sample derivation. CT, computed tomography; L1, lumbar 1; L2, lumbar 2; L3, lumbar 3; n, number; NHB, non-Hispanic Black; NHW, non-Hispanic White; SMI, skeletal mass index.

This retrospective, cross-sectional study included subjects from an overall lung cancer registry (n = 1251) of cases diagnosed or treated between 2014 and 2016. Half (n = 604) of the cases were reviewed, and 293 subjects were included based on a diagnosis of lung cancer, >/= 19 years of age, institutional diagnostic CT scan, and self-identified as NHB or NHW. Subjects were additionally excluded if they were missing data for L1, L2, or L3 SMI or the stage of cancer (n = 79). The registry was checked for accuracy, and duplicate entries or those with a restricted record were excluded from the analytic sample. The final dataset included 214 cases. See Figure 2 for details on exclusions and how the sample was derived.

### 5.2. Data Extraction

Electronic medical records (EMRs) were reviewed to collect demographic and clinical information relative to the date of cancer diagnosis. The data extracted included self-reported race, ethnicity, and sex; age, height, weight, tumor type, and the stage of cancer, which was categorized as early-stage (in situ, stage 1, and stage 2) versus late-stage (stages 3 and 4).

### 5.3. Image Analysis

Diagnostic CT scans were analyzed by board-certified diagnostic radiologists to extract cross-sectional images at L1, L2, and L3 using DICOM^®^ (Digital Imaging and Communications in Medicine) from CT scan protocols performed for diagnostic reasons as clinically indicated. Thoracic vertebral landmarks were not examined and thus outside the scope of this project. For subjects with more than one CT scan +/−45 days from the diagnosis date, the CT scan closest to the diagnosis date where all three lumbar vertebral landmarks (L1, L2, L3) could be obtained was chosen. For example, if the chest CT was closest to the diagnosis date but it only provided L1 extraction, then the whole-body CT or chest/abdominal/pelvis scan was chosen (still within 45 days of diagnosis). The cross-sectional images were uploaded into the Automated Body composition Analyzer using Computed tomography image Segmentation (ABACS+ by Voronoi Health Analytics Inc., Coquitlam, Canada) by trained research staff for the automatic segmentation of skeletal muscle and adipose tissues. ABACS+ is integrated into the SliceOmatic (TomoVision, Magog, QC, Canada) 5.0 software as a module. Skeletal muscle was identified using standard Hounsfield Units (HU) of −29 to +150 HU [28]. Images were manually examined after automatic analysis to validate the accuracy of skeletal muscle and adipose segmentation.

### 5.4. Primary Dependent Variables

The primary study outcome was sarcopenia defined as a binary variable. The Derstine et al. [11] criteria for sarcopenia diagnosis, based on two standard deviations below the healthy reference mean for the SMI, were used at L1, L2, and L3 to define sarcopenia status. These cut-offs are consistent with the European Working Group on Sarcopenia in Older People’s recommendation to use two standard deviations below the normative, healthy reference population mean to define sarcopenia [29]. The criteria include the following: L1 SMI as ≤25.9 cm^2^/m^2^ in females and ≤34.6 cm^2^/m^2^ in males; L2 SMI as ≤30.4 cm^2^/m^2^ for females and ≤40.1 cm^2^/m^2^ for males; and L3 SMI as ≤34.4 cm^2^/m^2^ for females and ≤45.4 cm ^2^/m^2^ for males. Once sarcopenia status was determined by sex, the groups were combined into the binary variable.

The skeletal muscle cross-sectional area (cm^2^) at L1, L2, and L3 was obtained from the image analysis results and normalized for height (m^2^) to calculate the SMI. We investigated L1 and L2 SMI because they are highly correlated with L3 SMI, which is the reference standard for body composition. As muscle strength was not measured in this study, we defined sarcopenia as the quantity of skeletal muscle assessed by a CT scan.

### 5.5. Statistical Analysis

Descriptive statistics (N [%] or mean [standard deviation]) were calculated, and statistical differences were estimated using an independent t-test for normally distributed continuous variables and Pearson’s chi-square test for categorical variables. Pearson’s correlation statistic was used to assess relationships between the L1 SMI and L3 SMI and between the L2 SMI and L3 SMI. The agreement of sarcopenia identification between L1 and L3 and between L2 and L3 was assessed via Cohen’s kappa coefficient and by the overall accuracy, sensitivity, specificity, and area under the curve. Statistical significance was defined as *p* < 0.05. SAS^®^ Studio (https://www.sas.com/el_gr/software/on-demand-for-academics.html, (accessed on 28 March 2024)), SAS^®^ OnDemand for Academics (Copyright© 2021, SAS Institute Inc., Cary, NC, USA) was used to conduct the statistical analysis. The university (Protocol #2020-0677) and hospital (ORA #18013002-IRB01) Institutional Review Boards approved this study.

## 6. Conclusions

The prevalence of sarcopenia in NHB and NHW individuals with lung cancer varied greatly depending on the vertebral landmark used for assessment. Because there was only weak and moderate agreement in the identification of sarcopenia at L1 and L2 compared to L3 and low sensitivity, our data indicate that using L1 or L2 may result in the misclassification of sarcopenia status in individuals with lung cancer. The misclassification of sarcopenia has clinical implications as a delay in nutrition-focused, physical activity, and medication interventions could impact lung cancer survival. Further research in larger studies using diverse populations is needed to determine the sensitivity and specificity of using L1 and L2 for sarcopenia identification in individuals with lung cancer.

## Figures and Tables

**Table 1:** Demographics of subjects with lung cancer overall and by race and sex (n = 214) ^1^.

	Overall Sample	*p*-Value	NHB (n = 107)	NHW (n = 107)	*p*-Value	Female (n = 107)	Male (n = 107)	*p*-Value
Age (years)	68.4 ± 9.9		67.4 ± 11.0	69.4 ± 8.5	0.13	69.4 ± 10.0	67.4 ± 9.7	0.13
Height (cm)	169.4 ± 10.5		169.0 ± 10.4	169.8 ± 10.7	0.57	162.5 ± 8.5	176.3 ± 7.4	<0.0001
Weight (kg)	75.6 ± 19.2		74.4 ± 20.0	76.7 ± 18.4	0.38	70.1 ± 18.0	81.1 ± 18.9	<0.0001
BMI (kg/m^2^)	26.3 ± 6.0		26.1 ± 6.4	26.5 ± 5.6	0.58	26.5 ± 6.3	26.1 ± 5.6	0.59
L1 SMI	37.1 ± 8.5		37.7 ± 9.2	36.5 ± 7.8	0.32	34.7 ± 7.3	39.5 ± 9.0	<0.0001
L2 SMI	39.4 ± 9.0		40.0 ± 9.5	38.9 ± 8.6	0.36	36.8 ± 8.1	42.1 ± 9.1	<0.0001
L3 SMI	42.8 ± 9.6		43.5 ± 10.0	42.2 ± 9.3	0.32	39.6 ± 8.1	46.1 ± 10.0	<0.0001
Sex								
Male	107 (50.0)	1.00	52 (48.6)	55 (51.4)	0.68			
Female	107 (50.0)		55 (51.4)	52 (48.6)				
Cancer Stage		<0.001			<0.001			0.48
Early-Stage	81 (37.9)		28 (26.2)	53 (49.5)		43 (40.2)	38 (35.5)	
Late-Stage	133 (62.1)		79 (73.8)	54 (50.5)		64 (59.8)	69 (64.5)	
Smoking History		<0.0001			0.84			0.01
Current/Former	192 (90.1)		96 (90.6)	96 (89.7)		90 (84.9)	102 (95.3)	
Never	21 (9.9)		10 (9.4)	11 (10.3)		16 (15.1)	5 (4.7)	

^1^ Values are means ± SDs or n (%). *t*-test used for continuous variables and Pearson’s chi-square test used for categorical variables. cm, centimeters; kg, kilograms; BMI, body mass index; m^2^, meters squared; L1, first lumbar; L2, second lumbar; L3, third lumbar; SMI, skeletal mass index; early-stage cancer, in situ, stage 1, and stage 2; late-stage cancer, stage 3 and stage 4; NHB, non-Hispanic Black; NHW, non-Hispanic White.

**Table 2:** Identification of sarcopenia at L1, L2, and L3 using sex-specific cut-off values [11] and demographics of subjects based on sarcopenia status (n = 214) ^1^.

	Cut-Off (≤25.9 cm^2^/m^2^ for Females and ≤34.6 cm^2^/m^2^ for Males) at L1			Cut-Off (≤30.4 cm^2^/m^2^ for Females and ≤40.1 cm^2^/m^2^ for Males) at L2			Cut-Off (≤34.4 cm^2^/m^2^ for Females and ≤45.4 cm^2^/m^2^ for Males) at L3		
	Sarcopenia ** (n = 42)	No Sarcopenia (n = 172)	*p*-Value	Sarcopenia ** (n = 67)	No Sarcopenia (n = 147)	*p*-Value	Sarcopenia ** (n = 85)	No Sarcopenia (n = 129)	*p*-Value
Overall Prevalence	19.6%	80.4%	<0.0001	31.3%	68.7%	<0.0001	39.7%	60.3%	0.003
Age (years)	68.6 ± 10.2	68.4 ± 9.8	0.87	68.8 ± 9.8	68.3 ± 9.9	0.74	70.0 ± 10.2	67.4 ± 9.5	0.05
Height (cm)	173.3 ± 9.1	168.4 ± 10.7	0.007	173.2 ± 10.0	167.6 ± 10.4	0.0003	172.1 ± 8.9	167.6 ± 11.1	0.001
Weight (kg)	60.8 ± 14.2	79.2 ± 18.6	<0.0001	66.4 ± 19.0	79.7 ± 17.9	<0.0001	70.1 ± 18.4	79.2 ± 19.0	<0.001
BMI (kg/m^2^)	20.1 ± 3.9	27.8 ± 5.4	<0.0001	21.9 ± 4.9	28.3 ± 5.3	<0.0001	23.6 ± 5.7	28.1 ± 5.5	<0.0001
Race/Ethnicity									
NHB	22 (52.4)	85 (49.4)	0.73	32 (47.8)	75 (51.0)	0.66	38 (44.7)	69 (53.5)	0.21
NHW	20 (47.6)	87 (50.6)		35 (52.2)	72 (49.0)		47 (55.3)	60 (46.5)	
Sex									
Female	12 (28.6)	95 (55.2)	0.002	24 (35.8)	83 (56.5)	0.005	31 (36.5)	76 (58.9)	0.001
Male	30 (71.4)	77 (44.8)		43 (64.2)	64 (43.5)		54 (63.5)	53 (41.1)	
Stage of Cancer									
Early-stage	10 (23.8)	71 (41.3)	0.04	20 (29.9)	61 (41.5)	0.10	29 (34.1)	52 (40.3)	0.36
Late-stage	32 (76.2)	101 (58.7)		47 (70.1)	86 (58.5)		56 (65.9)	77 (59.7)	

^1^ Values are means ± SDs or n (%). *t*-test used for continuous variables and Pearson’s chi-square test used for categorical variables. cm, centimeters; kg, kilograms; m^2^, meters squared; BMI, body mass index; early-stage cancer, in situ, stage 1 and stage 2; late-stage cancer, stage 3 and stage 4; NHB, non-Hispanic Black; NHW, non-Hispanic White. ** Sarcopenia strata were evaluated for differences by stage of cancer. Late-stage cancer was significantly higher in sarcopenia with *p* = 0.0007 at L1, *p* = 0.001 at L2, and *p* = 0.003 at L3. Sensitivity analysis for 1 cm below and above L1 published cut-off value resulted in sarcopenia prevalence range of 16.4–22.9%. Sensitivity analysis for 1 cm below and above L2 published cut-off value resulted in sarcopenia prevalence range of 27.6–36.0%.

**Table 3:** Level of agreement in identifying sarcopenia at L2 and L1 compared to L3 (n = 85 sarcopenia).

	Correctly Classified According to L3 n (%)	Area under Curve	Sensitivity %	Specificity %	Positive Predictive Value %	Negative Predictive Value %	Kappa	95% CI	*p*-Value
L2	178 (83.2)	0.81	68.2	93.0	86.6	81.6	0.64	0.53, 0.74	0.003
L1	163 (76.2)	0.71	44.7	96.9	90.5	72.7	0.46	0.34, 0.57	<0.0001

L1, lumbar 1; L2, lumbar 2; L3, lumbar 3. Sensitivity analysis = kappa ranges from 0.59 to 0.66 for values 1 cm below and above the cut-off value for L2; kappa ranges from 0.39 to 0.52 for values 1 cm below and above the cut-off value for L1.

**Table 4:** Demographics of three different sub-groups and comparison of Group 1 to Group 2 and 3 (n = 214) ^1^.

	Group 1: All Three Vertebral Landmarks Identified Sarcopenia (n = 35)		Group 2: All Three Vertebral Landmarks Identified No Sarcopenia (n = 119)		Group 3: Sarcopenia Identification Was Inconsistent (n = 60)		*p*-Value *	*p*-Value **
		*p*-Value		*p*-Value		*p*-Value		
Age (years)	67.9 ± 10.4		67.4 ± 9.7		70.8 + 9.5		0.76	0.18
Height (cm)	173.4 ± 9.2		167.1 ± 10.9		171.5 ± 9.5		0.003	0.35
Weight (kg)	59.0 ± 13.4		80.0 ± 18.5		76.6 ± 18.5		<0.0001	<0.0001
BMI (kg/m^2^)	19.5 ± 3.7		28.5 ± 5.3		25.9± 5.1		<0.0001	<0.0001
Race/Ethnicity								
NHB	19 (54.3)	0.61	63 (52.9)	0.52	25 (41.7)	0.20	0.89	0.23
NHW	16 (45.7)		56 (47.1)		35 (58.3)			
Sex								
Female	10 (28.6)	0.01	72 (60.5)	0.02	25 (41.7)	0.20	<0.001	0.20
Male	25 (71.4)		47 (39.5)		35 (58.3)			
Stage of Cancer								
Early-stage	9 (25.7)	0.004	50 (42.0)	0.08	22 (36.7)	0.04	0.08	0.27
Late-stage	26 (74.3)		69 (58.0)		38 (63.3)			

^1^ Values are means ± SDs or n (%). *t*-test used for continuous variables and Pearson’s chi-sqpleaseuare test used for categorical variables. cm, centimeters; kg, kilograms; m^2^, meters squared; BMI, body mass index; early-stage cancer, in situ, stage 1 and stage 2; kg, kilograms; late-stage cancer, stage 3 and stage 4; m^2^, meters squared; NHB, non-Hispanic Black; NHW, non-Hispanic White. * = comparison of Groups 1 and 2; ** = comparison of Groups 1 and 3.

## Data Availability

The datasets presented in this article are not readily available because the data are part of an ongoing study. Requests to access the datasets should be directed to the corresponding author.
